# Physiotherapy management of chemotherapy-induced peripheral neuropathy in Pretoria, South Africa

**DOI:** 10.4102/sajp.v76i1.1482

**Published:** 2020-10-06

**Authors:** Esther A. Niemand, Maria E. Cochrane, Carina A. Eksteen

**Affiliations:** 1 Department of Physiotherapy, Faculty of Health Care Sciences, Sefako Makgatho Health Sciences University, Pretoria, South Africa

## Abstract

**Background:**

The increase in newly diagnosed patients with cancer in South Africa and globally, may contribute to the increase in patients living with chemotherapy-induced peripheral neuropathy (CIPN). Chemotherapy-induced peripheral neuropathy negatively impacts on quality of life (QoL) during and post chemotherapy treatment. Physiotherapy management of CIPN helps patients to manage symptoms and improves function in activity- and participation-levels to ultimately improve QoL. However, little evidence exists regarding the type or combination of physiotherapy management strategies in South Africa.

**Objectives:**

The purpose of our study was to determine how the symptoms of CIPN were managed by physiotherapists in Pretoria, South Africa.

**Method:**

A quantitative, descriptive study design was used. Electronic questionnaires were distributed to physiotherapists who worked with cancer patients and who treated patients with CIPN.

**Results:**

Physiotherapists used massage, proprioceptive neuromuscular facilitation, sensory integration, activities of daily living training, postural drainage, lifestyle modifications; Bobath-, breathing-, stability-, stretching-, strengthening- and endurance-exercises; electrical stimulation, thermal modalities, transcutaneous electrical nerve stimulation, soft tissue mobilisation; muscle energy-, positional- and Mulligan-techniques in the management of CIPN.

**Conclusion:**

Cardiopulmonary therapy, therapeutic exercises, manual therapy and neuro-developmental techniques were used by physiotherapists in the management of CIPN. Almost half of the participants did not use electrotherapy techniques. Physiotherapy management strategies that are used in Pretoria are similar to published research.

**Clinical implications:**

Physiotherapists in Pretoria are managing CIPN according to international practices. However, studies to determine the effectiveness of the management strategies in a South African context should be conducted. Alternative management strategies, based on the pathophysiology of CIPN, should be explored.

**Keywords:**

physiotherapy; chemotherapy-induced peripheral neuropathy; intervention; management; cancer.

## Introduction

Worldwide it is projected that the incidence of cancer diagnoses will increase by 50% in the next 10 years (National Cancer Institute 2017). The National Cancer Institute (2017) projected that more than 60% of these diagnoses will affect Africa, South- and Central America and Asia. As early cancer diagnosis and better therapy becomes more prevalent, it is estimated that the number of cancer survivors will increase. In 2014 it was calculated that there was a 25% reduction in cancer mortality and this is expected to increase by 31% in 2026 (Rebecca, Kimberly & Ahmedin 2017).

Chemotherapy is one of the most common methods of treating cancer (Zajczkowska et al. 2019). Although this method of treatment benefits patients enormously, chemotherapy is also well known for its considerable side effects. Worldwide chemotherapy-induced peripheral neuropathy (CIPN) is one of the most common side effects of chemotherapy and can affect up to 68% of people receiving chemotherapy (Molassiotis et al. 2019).

The mechanism resulting in CIPN is intricate and multifactorial. Possible mechanisms involved in the development of CIPN include mitochondrial damage and oxidative stress, microtubule disturbances, damage to deoxyribonucleic acid and myelin of the peripheral nerves, immunological responses and neuroinflammation (Zajczkowska et al. 2019).

Chemotherapy-induced peripheral neuropathy can cause a severe and sudden onset of symptoms because of specific chemotherapeutic drugs (Zajczkowska et al. 2019). Symptoms are dose- and agent-dependent (Seretny et al. 2014). Clinical manifestation of CIPN presents itself in the form of sensory, motor and seldom autonomic symptoms with sensory symptoms being more common and developing first (Iźycki et al. 2016). Symptoms normally start distally and move more proximally with the feet being affected first. Sensory symptoms can include numbness, tingling, allodynia, hyperalgesia, paraesthesia, dysesthesia and altered vibration sense. Total sensory loss is possible in severe cases (Yoon & Oh 2018; Zajczkowska et al. 2019).

Motor symptoms are less common and present themselves as peripheral weakness, balance and gait impairments (Zajczkowska et al. 2019). Autonomic symptoms include orthostatic hypotension, gastrointestinal motility dysfunction and altered urinary and sexual function (Iźycki et al. 2016; Zajczkowska et al. 2019). Certain pre-existing comorbid conditions (previous neuropathy, poor renal function, paraneoplastic antibodies, human immunodeficiency virus, vitamin B deficiency, thyroid dysfunction and peripheral vascular disease) and risk factors (age, smoking) may contribute to worsening of CIPN symptoms (Zajczkowska et al. 2019).

Chemotherapy-induced peripheral neuropathy can influence activities of daily living and quality of life (QoL) of cancer survivors for up to 6 months after chemotherapy has been terminated (Seretny et al. 2014). When CIPN occurs during active chemotherapy it can lead to the discontinuation or reduction of the chemotherapy dose, which might result in the dosage not being effective in the management of cancer (Seretny et al. 2014). It is therefore important that physiotherapists explore new ways to effectively treat cancer survivors and manage the side effects that the survivors have to live with.

The physiotherapy management of CIPN has not been researched extensively. From an in-depth literature search, it was found that internationally, the most frequently used physiotherapy management strategies are: manual therapy techniques, such as massage (Brami, Bao & Deng 2016), neuro-developmental techniques, such as sensorimotor retraining (Duregon et al. 2018), cardiopulmonary therapy, such as breathing exercises and lifestyle modification (Derksen et al. 2017), therapeutic exercises (Kleckner et al. 2017) and electrotherapy modalities, such as diathermy, transcutaneous electrical nerve stimulation and photobiomodulation (PBM) (Freitas de Freitas & Hamblin 2016; Lindblad, Bergkvist & Johansson 2016).

Massage is reported to be the most commonly used manual therapy technique to improve CIPN-related symptoms (Brami et al. 2016). In their systematic review, Brami et al. (2016) found that the increased circulation associated with massage therapy results in a reduction of CIPN symptom severity and significant improvement of QoL (Brami et al. 2016).

The recommended weekly physical activity for adults is 150 min of moderate intensity exercise or 75 min of vigorous intensity exercise (Gibson, Wagner & Heyward 2017). A study conducted on colorectal cancer patients with CIPN found an indirect linear relationship between CIPN symptoms and the amount of physical activity the patients performed per week (Mols et al. 2014). The authors also found that patients who did not meet the weekly physical activity requirements reported significantly worse CIPN symptoms (irrespective of whether they were receiving chemotherapy or not) compared with patients who met the weekly physical activity requirements (Mols et al. 2014). Meeting the weekly physical activity requirements resulted in less intense CIPN symptoms and better QoL (Mols et al. 2014).

A combination of aerobic endurance, sensorimotor and strength training was used in a study by Streckmann et al. (2014) to improve CIPN symptoms in patients who received chemotherapy for lymphoma. Patients in this study received training for 36 weeks, twice per week, starting 24-h after cessation of chemotherapy. Although the authors report that the exercise regime was not suitable for all patients, they found that a variety of exercises reduced the symptoms of CIPN and improved QoL of the patients (Streckmann et al. 2014). Duregon et al. (2018) reported similar findings, during a systematic review, when a combination of sensorimotor-, strength- and endurance-exercises were prescribed for patients with CIPN. Despite the reported benefits of exercises, the authors recommended that exercises should be specifically tailored to each individual’s needs (Duregon et al. 2018). Symptomatic relief and QoL are not the only benefits of therapeutic exercises. Brayall et al. (2018) reported that patients living with CIPNs’ muscle strength, balance and function also improved with strength and endurance training.

Despite the reported benefits of therapeutic exercises, patients receiving neurotoxic chemotherapy resulting in CIPN symptoms may not be suitable candidates for this intervention. Kleckner et al. (2017) conducted a randomised control trial on 170 patients with CIPN symptoms as a result of neurotoxic chemotherapy. The experimental group was exposed to the ‘Exercise for Cancer Patient’ home programme consisting of moderate intensity walking and resistance exercises for 6 weeks (Kleckner et al. 2017). The participants in the control group continued with regular care. The results from their study indicate that the experience of CIPN symptoms worsened for all patients, because of the cumulative effect of the neurotoxic chemotherapy. However, the experimental group who performed the home programme showed smaller increases (*p* = 0.045) in symptom severity, compared with the control group (Kleckner et al. 2017).

Physiotherapists also use electrotherapy modalities, which provide sensory input, in the management of CIPN symptoms. Whole body vibration therapy is a relatively new therapy with potential benefits for CIPN patients. A phase 2 randomised exploratory study by Schönsteiner et al. (2017) indicate that whole-body vibration results in a significant improvement in coordination and fitness in patients with CIPN (Schönsteiner et al. 2017). Long-wave diathermy and interferential currents are also used to treat chronic CIPN symptoms (Lindblad et al. 2016). They found that long-wave diathermy and interferential current were equally effective in managing CIPN-related symptoms and that the duration and dosage do not determine the effectiveness of the modalities.

Photobiomodulation is another physical modality that has been investigated in the management of CIPN symptoms. Photobiomodulation is a light therapy from light emitting diodes, similar to laser (Freitas de Freitas et al. 2016). These studies have shown that PBM may facilitate the reduction of tumour size in rats, resolution of inflammatory processes, tissue healing and bone growth (Freitas de Freitas et al. 2016). In a double blind, randomised controlled trial an intervention group received 30 min of PBM three times per week, for 6 weeks. The patients who received PBM showed a significant reduction in CIPN symptoms (Argenta et al. 2017).

In South Africa, very little research has been done regarding the physiotherapy management of oncology patients with CIPN.

Thus the aim of our study was to investigate the physiotherapy management of CIPN in Pretoria, South Africa and compare whether it follows international management of CIPN.

## Research methods and design

In South Africa, Gauteng represents the largest number of oncology treatment centres (National cancer strategic framework for South Africa 2017–2022).

A quantitative, descriptive study utlised electronic questionnaires (PTPQ tool) uploaded onto Survey Monkey (an electronic questionnaire distribution website). The questionnaires were sent to physiotherapists in Pretoria who were known to work in oncology settings. The physiotherapists were recruited by identifying the different oncology treatment centres in Pretoria and then identifying the physiotherapy practices receiving referrals from those oncology centres. Physiotherapists were also recruited by word of mouth from physiotherapists working in the clinical field in Pretoria.

After identifying potential participants, an electronic information leaflet and link were sent to them via email and short message services. Participants who were willing to participate in the study provided consent (clicked the consent button) before online access to the questionnaire was granted. Follow-up reminders to complete the questionnaire were sent on a weekly basis. Access to the electronic link was granted to the physiotherapists for 4 months. After completion and submission of the questionnaire, the physiotherapists could not access the link.

The PTPQ is a survey tool that was developed to comprehensively describe the practice of physiotherapy in various clinical settings. The questionnaire consists of five different components, namely: general information and demographics; practice profile; treatment preferences; bases for clinical work; and bases for education or research work. For our study, physiotherapists were asked to complete the ‘treatment preferences’ component of the PTPQ for CIPN management specifically. The content validity of the PTPQ was established based on input from an expert panel (Dizon, Grimmer-Somers & Kumar 2011). The internal consistency and reliability for the PTPQ is high (Chronbach’s α = 0.90) and (0.89) (Dizon et al. 2011). A qualitative analysis showed that the PTPQ is easy to use and the format is concise and satisfactory (Dizon et al. 2011).

The collected data were analysed using descriptive analysis with SPSS version 24.0, in order to meet the aims and objectives of the study.

### Ethical consideration

Clearance was obtained from Sefako Makgatho Health Sciences University Ethics Committee, all institutions where the study was conducted, each individual participant and the data collection tool developers. Ethical Clearance Number: SMUREC/H/265/2017:PG, 5 October 2017

## Results

**FIGURE 1:**
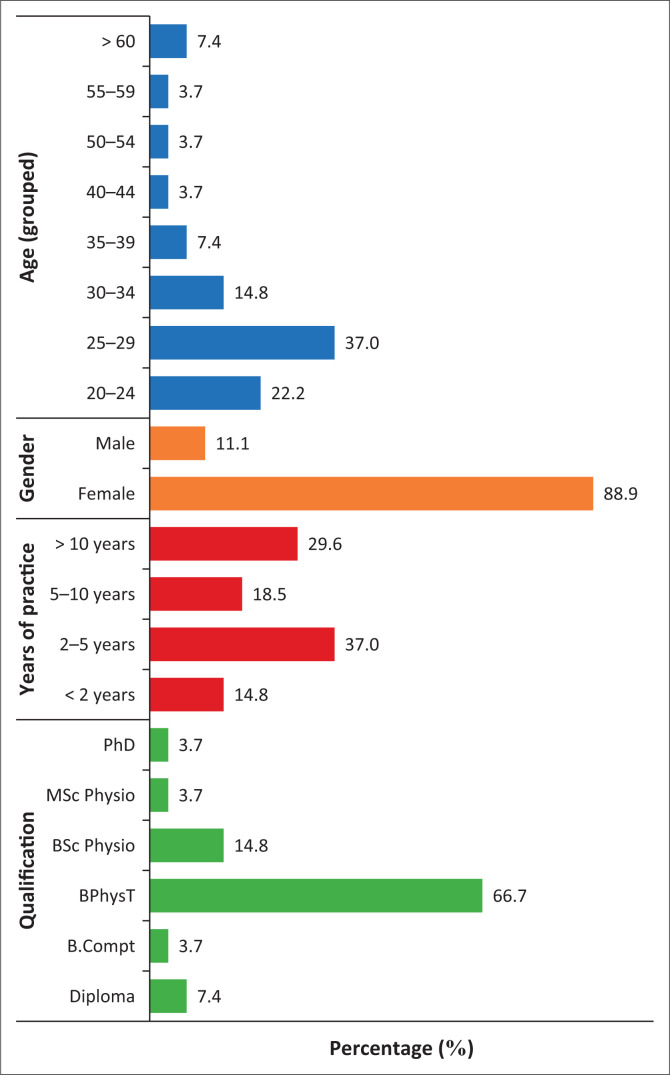
Descriptive statistics of participants.

Twenty-seven (27) physiotherapists participated and their ages ranged from 23 to 71 (M 32.85; SD 12.703). The majority of the participants (*n* = 24; 88.89%) were female. Ten (37%) indicated that they have been in practice between two and five years. Eighty two percent (*n* = 22) had either a Bachelor of Science in Physiotherapy or a Bachelor in Physiotherapy degree (Figure 1). Fifty two percent (*n* = 14) worked in a general physiotherapy practice. Three (11.11%) of the 27 participants worked exclusively with oncology patients, the rest (*n* = 24; 88.89%) treated oncology patients on an irregular basis.

In order to establish the management strategies that were used for patients with CIPN, the participants were asked to indicate the techniques that they use under the following headings: manual therapy techniques, neuro-developmental techniques, cardiopulmonary therapy, therapeutic exercises, electrotherapy techniques and other. Participants could indicate that they use more than one technique (Table 1).

**TABLE 1:** Descriptive statistics for the management strategies of chemotherapy-induced peripheral neuropathy by physiotherapists.

Technique	No. of physiotherapists	
	*n*	%
**Manual therapy techniques**		
Soft tissue mobilisation	18	66.67
Muscle energy techniques	7	25.93
Positional techniques	7	25.93
Mulligan’s techniques	4	14.81
Massage	7	25.93
No manual therapy techniques	3	11.11
Other manual therapy techniques	1	3.7
**Neuro-developmental techniques**		
Bobath exercises	6	22.22
Brunnstrom exercises	0	-
Proprioceptive neuromuscular facilitation	17	62.96
Sensory integration	18	66.67
No neuro-developmental techniques	2	7.41
Other neuro-developmental techniques	0	-
**Cardiopulmonary therapy**		
Breathing exercises	15	55.56
Activities of daily living	15	55.56
Postural drainage	6	22.22
Lifestyle modifications	13	48.15
No cardiopulmonary techniques	3	11.11
Other cardiopulmonary techniques	0	-
**Therapeutic exercises**		
Stability exercises	21	77.78
Stretching exercises	16	59.26
Strengthening exercises	18	66.67
Endurance exercises	16	59.26
No therapeutic exercises	0	-
Other therapeutic exercises	0	-
**Electrotherapy techniques**		
Ultrasound	2	7.41
Electrical stimulation	7	25.93
Infrared	0	-
Hot or cold modalities	6	22.22
Diathermy	0	-
Transcutaneous electrical nerve stimulation	4	14.81
Laser	3	11.11
No electrical therapy techniques	13	48.15
Other electrical therapy techniques	0	-
**Any other techniques**		
Nerve gliding	2	7.41
Balance retraining	2	7.41

From Table 1, it can be seen that physiotherapists in Pretoria favoured the following treatment techniques in the management of CIPN: stability exercises (77.78%), soft tissue mobilisation (66.67%), sensory integration (66.67%), strengthening exercises (66.67%), proprioceptive neuromuscular facilitation (62.96%) stretching endurance-exercises (59.26%) breathing exercises and activities of daily living training (55.56%).

## Discussion

It is projected that in the near future the leading cause of morbidity in low and middle income countries, like South Africa, will be cancer. Cancer diagnoses in South Africa are expected to rise by 46%, to 112 921 new cases each year, by 2030 (Singh et al. 2015). With the growing rate of new cancer diagnoses and increased survival nationally and globally, identification and management of the side effects of cancer treatment (especially with regard to chemotherapy) has to be prioritised. The role and importance of physiotherapy in the management of cancer and the associated symptoms of cancer treatment, such as CIPN, has been acknowledged by the South African Department of Health (Breast Cancer Control Policy 2017). However, despite the increased number of patients surviving cancer and the acknowledgement that physiotherapy plays a role in the management of these patients, the small sample size in our study may be interpreted as being that very few physiotherapists in Pretoria solely treat the side effects with which oncology patients present. Our results indicate that the majority of physiotherapists who manage patients with CIPN work in general private practices and that rehabilitation of oncology patients is not their primary focus. This may be because physiotherapists in South Africa can join a special interest group, but do not have the option to specialise and market themselves in a specific field. Many physiotherapists may also treat conditions that resulted from CIPN, but may not realise that they are also assisting in the management of cancer-related side effects. It is also possible that medical personnel working in oncology are unaware of the benefits that physiotherapists can contribute to the treatment of cancer-related side effects and therefore do not consider physiotherapy management.

The physiotherapist’s role in the management of patients with cancer has been identified as: decreasing length of stay in acute facilities, improving functional ability, improving lymphodoema management, improving local and general exercise capacity and shortening the recovery time after surgery and improving QoL (Bernardo-Filho et al. 2014). The role of physiotherapists in the treatment of CIPN is often overlooked and has not been included in general oncology studies. However, Jung, Rein and Fuchs (2016) clearly indicated the critical importance of physiotherapy for patients who suffer from CIPN.

Manual therapy techniques, neuro-developmental techniques, cardiopulmonary therapy and therapeutic exercises were predominantly used in the management of CIPN by our participants. Almost half indicated that they did not use electrotherapy techniques at all in the management of CIPN. This is in contrast to what is reported in the literature where the use of long-wave diathermy, interferential currents (Lindblad et al. 2016) and PBM (Argenta et al. 2017) are reported to be beneficial in the treatment of CIPN.

The physiotherapy management that was most frequently implemented was: stability exercises, soft tissue mobilisation, sensory integration, strengthening exercises, proprioceptive neuromuscular facilitation (62.96%) stretching and endurance exercises, breathing exercises and activities of daily living training.

These results are similar to the management of CIPN in breast cancer patients reported by Ceprnja and Maka (2017). They found that the majority of physiotherapists prescribed different types of exercise as the first-line treatment for CIPN. They also indicated that 60% of physiotherapists use soft tissue techniques for the management of CIPN and only 5% of their participants (Australian physiotherapists) used electrotherapy modalities in the management of CIPN.

The neuro-developmental modalities that were mostly implemented by physiotherapists were: proprioceptive neuromuscular facilitation and sensory integration. Streckmann et al. (2014) reported the benefits of sensorimotor training, like postural stabilisation and sensory integration activities, to improve patients who live with CIPN’s QoL. These sensorimotor training techniques were performed in combination with aerobic and strength training. Duregon et al. (2018) found that a combination of sensorimotor-, strength- and endurance-training helped to improve the QoL of patients with CIPN. There is no published literature that advocates the use of proprioceptive neuromuscular facilitation per se for the management of CIPN symptoms or improvement of QoL of patients with CIPN.

Cardiopulmonary therapy in the management of CIPN was also used with the most frequently used techniques being: breathing exercises, activities of daily living training and lifestyle modifications. Galantino et al. (2012) report that patients with CIPN who received breathing exercises reacted with a reduction in the symptoms they experienced. Other than this study by Galantino et al. (2012), very little research exists on the effectiveness of cardiopulmonary therapy for the management of CIPN and improvement in QoL in patients with CIPN.

Locally and internationally, exercise prescription for the management of CIPN symptoms and improvement of QoL is the most frequently and effectively implemented physiotherapy modality. However, exercises should not be prescribed in isolation. The inclusion of different forms of therapeutic exercises in an exercise programme has been found to be more effective than the performance of a single type of exercise (Brayall et al. 2018; Duregon et al. 2018; Kleckner et al. 2017). The starting position in which exercises are prescribed should also be considered. Closed kinematic chain exercises significantly improve patients with CIPN’s balance and QoL (Brayall et al. 2018). The individualisation of exercise programmes has also been emphasised (Streckmann et al. 2014). These authors found that intensive exercise programmes are not suitable for all CIPN patients and will affect QoL adversely (Streckmann et al. 2014). All the physiotherapists who participated in our study indicated that they use therapeutic exercises for the management of CIPN which is similar to international standards and current best practices.

It is noticeable from our study and the literature that physiotherapists use a vast variety of treatment techniques when it comes to the management of CIPN. The development of CIPN is intricate and multifactorial (Zajczkowska et al. 2019), resulting in several possible sensory, motor and autonomic symptomes (Iźycki et al. 2016). These symptoms can develop at different stages of the treatment process (Iźycki et al. 2016) resulting in the patient possibly presenting with a different clinical picture between treatment sessions. Participants were not asked which treatment technique they use at a specific stage of the development of CIPN. This might be the reason for the vast variety of treatment techniques used to treat CIPN. It is important that physiotherapists clinically reason the use of specific treatment techniques in the treatment of CIPN (Duregon et al. 2018).

**FIGURE 2:**
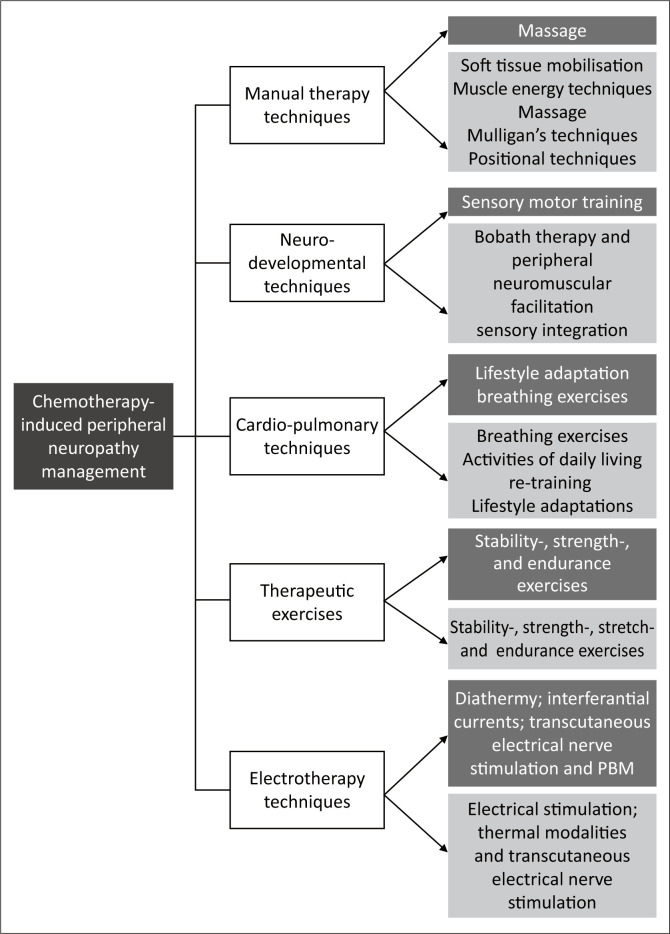
Summery of physiotherapy management modalities.

Figure 2 illustrates a summary of the physiotherapy modalities that were identified in the literature (indicated in dark grey) and what were identified in our study (indicated in light grey).

## Conclusion

Physiotherapy management strategies that are used in Pretoria, South Africa are similar to those in other studies. Because of the geographical restrictions of our study and the fact that the majority of participants did not exclusively treat oncology patients, our results may not be a true reflection of the physiotherapy management strategies of CIPN in South Africa. Future studies should be conducted to determine whether the physiotherapy management strategies that are used in Pretoria, South Africa, improve the QoL of patients living with CIPN and also to explore alternative management strategies.
